# A polygenic and family risk score are both independently associated with risk of type 2 diabetes in a population-based study

**DOI:** 10.1038/s41598-023-31496-w

**Published:** 2023-03-23

**Authors:** Elena Duschek, Lukas Forer, Sebastian Schönherr, Christian Gieger, Annette Peters, Florian Kronenberg, Harald Grallert, Claudia Lamina

**Affiliations:** 1 Institute of Genetic Epidemiology, Medical University of Innsbruck, Innsbruck, Austria; 2 Institute of Epidemiology, Helmholtz Zentrum München, German Research Center for Environmental Health, Neuherberg, Germany; 3 German Research Center for Cardiovascular Research (DZHK e.V.), Partner Site Munich Heart Alliance, Munich, Germany; 4 Research Unit of Molecular Epidemiology, Helmholtz Zentrum München, German Research Center for Environmental Health, Neuherberg, Germany; 5 Chair of Epidemiology, Ludwig-Maximilians Universität München, Munich, Germany

**Keywords:** Genetics, Diseases

## Abstract

The availability of polygenic scores for type 2 diabetes (T2D) raises the question, whether assessing family history might become redundant. However, family history not only involves shared genetics, but also shared environment. It was the aim of this study to assess the independent and combined effects of one family risk score (FamRS) and a polygenic score (PGS) on prevalent and incident T2D risk in a population-based study from Germany (n = 3071). The study was conducted in 2004/2005 with up to 12 years of follow-up. The FamRS takes into account not only the number of diseased first grade relatives, but also age at onset of the relatives and age of participants. 256 prevalent and additional 163 incident T2D cases were recorded. Prevalence of T2D increased sharply for those within the top quantile of the PGS distribution resulting in an OR of 19.16 (*p* < 2 × 10^–16^) for the top 20% compared to the remainder of the population, independent of age, sex, BMI, physical activity and FamRS. On the other hand, having a very strong family risk compared to average was still associated with an OR of 2.78 (*p* = 0.001), independent of the aforementioned factors and the PGS. The PGS and FamRS were only slightly correlated (r^2^_Spearman_ = 0.018). The combined contribution of both factors varied with varying age-groups, though, with decreasing influence of the PGS with increasing age. To conclude, both, genetic information and family history are relevant for the prediction of T2D risk and might be used for identification of high risk groups to personalize prevention measures.

## Introduction

Type 2 diabetes (T2D) is a growing problem in modern societies. The proportion of affected individuals has been gradually increasing for the past few decades^1^. In addition to lifestyle factors such as obesity, disadvantageous eating behaviours, and a lack of physical activity, it has been shown that genetics plays a big role too in susceptibility to T2D^2^. Several studies investigated the heritability of T2D resulting in estimates between 20 and 80% with a median of 40%^3–5^.

This rise in prevalence needs to be addressed and halted by improving effectiveness of prevention measures. It has been suggested that those measures should be more personalized and especially target high risk groups^6^. Knowledge on which individual is at high genetic risk to develop T2D in the future might be one of the key points.

A cluster of T2D cases in a family can be an indication for a high genetic risk for T2D. Having first degree relatives with T2D increased the risk of individuals to develop T2D themselves by three times compared to individuals without a positive family history^7^. The number of affected parents, but also the age at which the parents were diagnosed greatly influenced the estimation of a person’s diabetes risk^8^. Therefore, we used a family risk score incorporating not only the number of affected parents and siblings, but also weights for their disease onset and takes into account the participants’ age^9^.

A more puristic approach to a persons’ genetic risk lies in the measurement of genotypes and calculation of genetic risk scores. With genetic information becoming more easily available, the finding that genes seem to play a role in T2D has led to studies investigating polygenic scores (PGS). Khera et al.^10^, showed that such a genome-wide PGS can be used to identify more than 3% of the population with a more than threefold risk for T2D than the remaining part of the general population^10^. Thus, such scores might help to target high-risk individuals and offer personalized prevention measures.

We have recently shown that both family history, represented as the aforementioned family risk score^9^, and genome-wide polygenic scores are independent predictors for the risk of stroke^11^ and myocardial infarction^12^. There are no such studies for T2D, yet.

Therefore, we aimed to investigate the independent and combined effect of a genome-wide polygenic score (PGS) and a family risk score (FamRS) on prevalent and incident T2D risk in the population-based KORA F3 study.

## Methods

### Study population

The KORA studies are a series of population-based studies that were conducted in the city of Augsburg, Germany, and surrounding counties. KORA F3 was performed in 2004/2005, which was the baseline visit for the present investigation. Participants were chosen randomly, stratified for 10 year age-groups and sex, resulting in 3184 participants. The analysis dataset, with available genotype data and variables of interest (family history and prevalent and incident diabetes) comprised of 3071 participants. The applied study methods included a standardized computer-assisted interview, a physical examination and a blood sampling at baseline^13,14^. The participants were followed-up for mortality and morbidities (including diabetes status) until 2016. The median follow-up time was 11 years with 25,363 person years.

The study was approved by the Ethics Committee of the Bavarian Medical Association (Bayerische Landesärztekammer), all research was performed in accordance with relevant guidelines/regulations and written informed consent was obtained from all participants.

### Definition of diabetes

All information given in the interview at baseline (2004/2005) on diabetes and types of diabetes were validated by patient-records and/or by the patients’ general practitioners, resulting in 256 participants with type 2 diabetes. Age at diagnosis was self-declared in a standardized interview. The incident diabetes cases (n = 163) were defined as those who did not have T2D at the time of the baseline KORA F3 study (2004/2005), but who developed it in the timespan between KORA F3 and the follow-up in 2016. For incident cases, diabetes status, type of diabetes and age at diagnosis was validated by medical charge review or contactin the respionsible physician. This information was not derived for the 256 prevalent cases and was additionally missing for 280 participants. Therefore, the analysis dataset for the incident analysis comprised of 2535 participants.

### Definition and calculation of the FamRS

In order to obtain the FamRS, participants were asked the following questions for both parents and all siblings in a standardized interview: “Does or did your … (e.g. father) have one of the following diseases?” with diabetes as one of the diseases in the list. No specification of diabetes subtypes was made. If the question for diabetes was answered with yes, it was followed-up by the question, if it was “before the age of 60”, “at the age of 60 or later” or “age unknown” to obtain an age-dependent weight. The FamRS score was then obtained by comparing this weighted observed number of affected family members with the number that would have been expected giving the number of relatives and age of the respective participant. More details and the algorithm can be found in^9,15^ and the Supplementary Material.

The calculated FamRS was then used both as a continuous variable as well as a categorical variable. For the categorical variable, the following categories were applied^9^: “average risk” for FamRS ≤ 0.5, “positive risk” for 0.5 < FamRS ≤ 1, “strong positive risk” for 1 < FamRS ≤ 2 and “very strong positive risk” for FamRS > 2. For most analyses, categories “positive risk” and “strong positive risk” were joined due to low numbers of participants in the “positive risk” group.

### Genotyping and calculation of the PGS

In the KORA-F3 study, genotypes were derived using the Illumina Omni 2.5 and Illumina Omni Express array. Genotypes were imputed using the HRC reference panel^16^ and the Michigan imputation Server^17^. With that method, about 40 million SNPs over the whole genome are available for each of the KORA-F3 study participants. The PGS for T2D that was used in this study was developed and validated by Khera et al.^10^ in more than 400,000 European samples (UK Biobank). The weights for the 6,917,436 Mio SNPs included in this PGS were derived from the Polygenic Score (PGS) Catalog^18^ as score number “PGS000014” and calculated using PGS-Calc (https://github.com/lukfor/pgs-calc) by summing up the product of all imputed genotype scores with their respective weights.

### Statistical analysis

Several logistic regression models were performed with both prevalent and incident T2D as outcome. FamRS and PGS were analyzed individually using three adjustment models each: (a) unadjusted model, (b) adjusted for sex, age, BMI and physical activity (PA) and c) additionally adjusting for PGS (in the FamRS model) or FamRS (in the PGS model). All covariates were assessed at the baseline visit. Since FamRS is highly right-skewed, it is not considered as a continuous variable, but was divided into three categories (average risk, positive or strong positive (FamRS 2) or very strong positive risk (FamRS 3)). PGS was considered as a continuous variable with the effect size given for one standard-deviation (sd) increase (1 sd of PGS = 0.123). Since a non-linear relationship of PGS with the prevalence of T2D was observed, the ORs are also given for several upper thresholds of the PGS (20%, 10%, 5% or 1%). Logistic regression models were also stratified for 10-year age-groups. Due to lower numbers of cases in these subgroups, these models are not adjusted for any other covariates except FamRS and PGS, which were included mutually. Further, FamRS was only divided into two categories in this analysis: average and above average (FamRS > 0.5). Since there were only 6 prevalent cases in the 35–44 y group, this age-group was omitted from this analysis. To account for the limited follow-up time for those, who died during follow-up, inverse probability of censoring weighting was applied for the incident analyses using package riskRegression in R. For each participant, the inverse of the probability of not being censored is calculated using a Cox-model. This is then used as weights in the logistic regression models.

To evaluate best thresholds for discrimination, ROC curves were used and AUC calculated (using function pROC in R). The predictive capacity of PGS and FamRS was determined by continuous Net Reclassification Index (NRI) and Integrated Discrimination Improvement (IDI) using the function improveProb in package Hmisc. Both adjustment models—adjusted for age, sex, BMI, physical activity plus an additional model including either PGS or FamRS – were used as baseline models to compare with. All analyses were performed using the programme R version 4.1.0. *p*-values < 0.05 were considered to be statistically significant.

## Results

### Population characteristics

Descriptive statistics can be found in Table 1. The KORA-F3 analysis dataset consists of 3071 participants, 1575 of which (51.2%) are female, in the age range 35–84 years (mean age 57.4 years) and mean BMI of 27.66. 51.7% were physically active (defined as regular activity for ≥ 1 h per week). At baseline, there were 256 participants with T2D (8.3%), 10 with type 1 diabetes and 9 other or unclear types. From the 2535 participants without prevalent T2D included in the Follow-up analysis, 163 developed T2D after the baseline visit. 35 of those participants with incident T2D and 264 without incident T2D died within the Follow-up period. Both absolute and relative numbers of T2D increased with age at baseline (Supplementary Table S1). The average age at onset (i.e. age at diagnosis as given in the interview) for participants with T2D at baseline was 58 years. Average age at baseline for those participants was 67 years. For incident T2D cases, average age of diagnosis was 67.5 years.

**Table 1:** Characteristics of the KORA F3 study (n = 3,071).

Variable	
Age (in years)	57.4 ± 12.88 [46.0, 57.0, 67.0]
Women	1575 (51.2%)
Anthropometric measurements	
Weight (in kg)	77.93 ± 15.00 [66.90,76.80,87.60]
BMI	27.66 ± 4.61 [24.42, 27.13, 30.28]
Height (in cm)	167.7 ± 9.46 [160.5, 167.4, 174.6]
Waist circumference (in cm)	94.93 ± 13.15 [85.6, 95.10, 103.60]
Hip circumference (in cm)	106.9 ± 8.98 [100.9, 105.6, 111.8]
Waist-Hip-Ratio	0.8867 ± 0.085 [0.8240, 0.8890, 0.9470]
Lifestyle	
Physically active	1589 (51.7%)
Categorizations of physical activity	
Regularly > 2 h per week	688 (22.4%)
Regularly active for ~ 1 h per week	901 (29.3%)
Irregularly active for ~ 1 h per week	460 (15%)
Little to no physical activity	1,022 (33.3%)
Smoking categories	
Regular smoker	490 (16.0%)
Irregular smoker	52 (1.7%)
Ex-smoker	1078 (35.1%)
Never-smoker	1283 (41.8%)
Family history for diabetes	
T2D in any parent	777 (25.5%)
Categorization of Family Risk score FamRS	
Average	2582 (84.7%)
Positive FamRS	343 (11.2%)
Strong positive FamRS	125 (4.1%)
Blood values	
Glucose (mg/dl)	107.2 ± 32.27 [91.0, 100.0, 113.0]
HbA1c-values (%)	5.369 ± 0.54 [5.100, 5.300, 5.500]
Total cholesterol (mg/dl)	218.3 ± 39.94 [191.0, 216.0, 243.0]
HDL cholesterol (mg/dl)	58.77 ± 17.15 [46.00, 56.00, 69.00]
LDL cholesterol (mg/dl)	128 ± 32.63 [105, 127, 148]
Triglycerides (mg/dl)	165.2 ± 126.05 [88.0, 136.0, 201.0]
Diabetes	
Number of prevalent Type 2 cases, n (%)	256 (8.3%)
Number of incident Type 2 cases, n (%)	163 (5.3%)

Continuous variables are shown as mean ± SD and [25%, 50%, 75%] percentile; Categorical variable in n (%).

### Distribution of the FamRS

**Figure 1:**
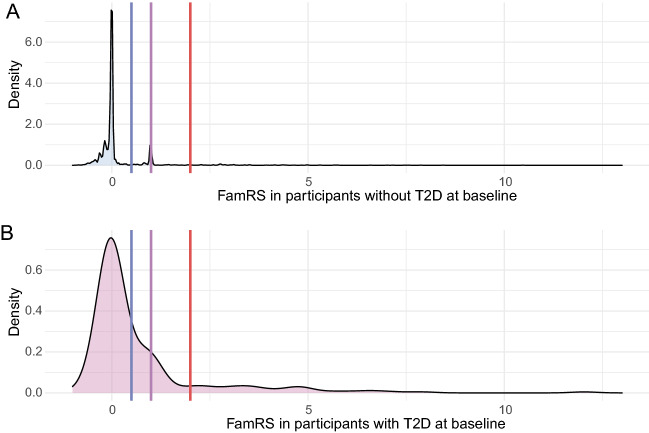
Density plot showing the FamRS distribution in participants without (lightblue, panel **A**) and with T2D (pink, panel **B**) at baseline; the lines depict the thresholds of the FamRS categories: positive family risk (blue line), strong positive family risk (purple), very strong positive family risk (red line).

Supplementary Fig. S1 shows the increasing proportion of T2D for increasing number of relatives affected by diabetes. The FamRS distribution markedly differs between participants with or without T2D at baseline with a high peak at 1 for non-cases and a shift to higher values for cases (Fig. 1). For the incident cases, there is an overrepresentation of peaks above zero compared to those participants not developing diabetes during follow-up (Supplementary Fig. S2). Supplementary Table S1 further shows an increase of prevalent and incident T2D over the FamRS risk categories. Supplementary Fig. S3 gives the distribution of the FamRS in T2D cases stratified in 10-year age-groups, showing a decreasing trend for increasing age. Therefore, FamRS seems to be highest for younger participants with T2D.

### Distribution of the PGS

**Figure 2:**
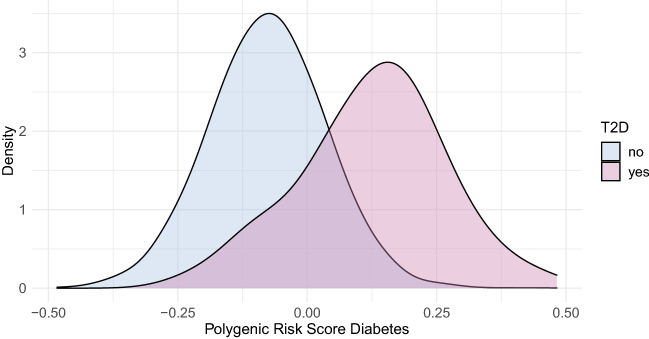
Density plot showing the PGS distribution in participants without (lightblue) and with T2D (pink) at baseline.

The overall distribution of the PGS in the KORA F3 study is given in Supplementary Fig. S4 together with corresponding distributions in 5 different populations from the 1000 Genomes reference population^19^. It closely matches the distribution of the Europeans, as expected. A clear right shift of the distribution can be observed in participants with T2D compared to those without T2D at baseline (Fig. 2), in consequence leading also to an increase of T2D cases for increasing percentile groups of the PGS (Supplementary Table S1). For incident cases, the shift is also notable but less pronounced (Supplementary Fig. S5). As was observed for the FamRS, the PGS is decreasing with age at baseline for T2D cases (Supplementary Fig. S6). Supplementary Fig. S7 depicts the prevalence of T2D in percentile groups of the PGS (in 5% groups from 0–5 to 95–100%). Up until the 75%-mark, there was no obvious difference in prevalence between the groups, with a steep increase above the 85%-percentile.

### Relationship between FamRS and PGS

The PGS was found to increase with increasing number of family members affected by T2D (Supplementary Fig. S8), which is only diluted in those with more than 6 affected family members. This group only consists of three participants, though.

**Figure 3:**
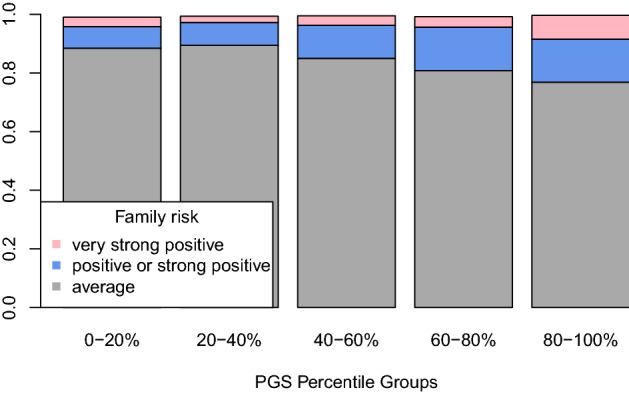
Relative amount of individuals in each FamRS category across the PGS percentile groups.

The relative amount of individuals with positive to very strong positive familial risk increases with increasing PGS-percentiles (Fig. 3). The frequency of individuals with more than average FamRS is more than doubling from about 10% in the lowest 20% of the PGS distribution to 22% in the highest 20%.

All in all, both measures are only slightly, although significantly, correlated (r^2^_Spearman_ = 0.018, *p* = 5 × 10^–14^).

### Results of logistic regression models

Table 2 shows the results of the logistic regression models of continuous PGS on both prevalent and incident T2D risk. The effect of the PGS was quite stable and highly significant across all models, with ORs ranging from 5.95 to 6.21 for prevalent and 1.66 and 1.68 for incident cases (per 1 sd increase). Due to the nonlinear and steep increase of T2D prevalence especially in the upper 20% of the PGS distribution (Supplementary Fig. S7), the logistic regression was repeated for several upper thresholds for PGS, with the respective remaining of the distribution as the reference category. The ORs increased from 19 to 47 for the upper 20% to 5% of the PGS distribution, even adjusted for age, sex, BMI, PA and FamRS (Table 3). For incident cases, a significant association can be observed for all presented top percentiles, with an OR of about 5 for the highest 5% of the PGS distribution compared to the remainder.

**Table 2:** Results of Logistic regression models of the effect of PGS on risk of prevalent and incident diabetes.

	Outcome: Prevalent T2D			Outcome: Incident T2D		
	OR*	CI (95%)	*p*-value	OR*	CI (95%)	*p*-value
Unadjusted	5.95	[4.97–7.20]	< 2 × 10^–16^	1.66	[1.38–1.99]	4.49 × 10^–8^
Adjusted for Age + Sex + BMI + Physical activity	6.35	[5.18–7.89]	< 2 × 10^–16^	1.68	[1.39–2.04]	1.08 × 10^–7^
Adjusted for Age + Sex + BMI + Physical activity + FamRS	6.21	[5.06–7.74]	< 2 × 10^–16^	1.67	[1.37–2.03]	2.63 × 10^–7^

* OR are given for increase in 1 SD of PGS (0.123).

**Table 3:** Results of Logistic regression models of the effect of PGS cut-offs on risk of prevalent and incident diabetes.

	Outcome: Prevalent T2D			Outcome: Incident T2D		
	OR*	CI (95%)	*p*-value	OR*	CI (95%)	*p*-value
Upper 20% of PGS	19.16	[13.65–27.27]	< 2 × 10^–16^	2.16	[1.45–3.18]	1.21 × 10^–4^
Upper 10% of PGS	32.09	[22.21–47.05]	< 2 × 10^–16^	2.95	[1.68–4.99]	9.35 × 10^–5^
Upper 5% of PGS	47.03	[29.69–76.26]	< 2 × 10^–16^	4.94	[2.00–11.35]	2.67 × 10^–4^

All models were adjusted for age, sex, BMI, physical activity and FamRS.

*OR is given for those above this cut-off with the remainder of the population as the reference.

The respective results for FamRS are given in Table 4. Having a positive or strong positive family risk is significantly associated with T2D risk with an OR of 2.08, with more than double the risk (OR 4.73) for a very strong positive family risk. The ORs do not markedly change, if the model is adjusted for age, sex, BMI and PA. It is attenuated, though, but still significant, when the PGS is additionally included in the model.

**Table 4:** Results of Logistic regression models of the effect of FamRS on risk of prevalent and incident diabetes

	FamRS categories	Outcome: Prevalent T2D			Outcome: Incident T2D		
		OR*	CI (95%)	*p*-value	OR*	CI (95%)	*p*-value
Unadjusted	FamRS 2	2.08	[1.45–2.92]	4.05 × 10^–5^	1.40	[0.85–2.19]	0.165
	FamRS 3	4.73	[3.04–7.20]	1.77 × 10^–14^	3.46	[1.85–6.09]	5.89 × 10^–5^
Adjusted for Age + Sex + BMI + Physical activity	FamRS 2	2.47	[1.67–3.61]	4.30 × 10^–6^	1.60	[0.95–2.58]	0.062
	FamRS 3	4.33	[2.65–6.95]	2.17 × 10^–9^	3.74	[1.94–6.82]	3.49 × 10^–5^
Adjusted for Age + Sex + BMI + Physical activity + PGS	FamRS 2	1.67	[1.01–2.71]	0.041	1.45	[0.86–2.35]	0.149
	FamRS 3	2.78	[1.49–5.09]	0.001	3.47	[1.79–6.35]	1.07 × 10^–4^

*ORs are given for FamRS categories “positive and strong positive family risk” (FamRS 2) and “very strong positive family risk” (FamRS 3) compared to average family risk.

The effect on incident diabetes was more robust with hardly a difference in ORs between the adjustment models. However, only very strong positive family risk remains significantly associated with an OR of 3.47 in the fully adjusted model. Simplifying the family history to “parents with T2D yes/no” also yields significant association with T2D risk (Supplementary Table S2). The ORs are in about the same range as the one for (strong) positive family risk (OR ranging from 2.14 to 2.68 in the prevalent models and 1.49–1.67 in the incident models), therefore missing the higher risk for those with very strong positive FamRS.

Since Supplementary Figs. S3 and S6 showed decreasing FamRS and PGS with increasing age, it was also evaluated, whether the effect of both scores changed with age. Therefore, logistic regression models were further stratified into 10y age-groups. The effect of PGS is slightly higher for those being 45–64 years at baseline than for older participants with no significant difference, though. The effect also doesn’t change, if additionally adjusted for FamRS (Supplementary Fig. S9, panel B). In contrast, the direction of interaction between age and FamRS on T2D changes, when PGS is taken into account (Supplementary Fig. S9, panel A). In line with the PGS, the OR for FamRS decreases slightly with increasing age in the unadjusted model. It increases, though, especially for the highest age-group, when PGS is adjusted for.

### Discrimination and reclassification measures

When the PGS is used as a classifier for prevalent diabetes, the area under the curve (AUC) amounts to 0.869, and the calculated best threshold is estimated to be 0.016 (~ 75% percentile), (Supplementary Table S3. For incident cases, AUC is markedly lower, but still significantly different from 0.5.

For FamRS the AUC for prevalent cases is 0.617 with the best threshold at 0.109. This threshold reflects the boundary of the high peak at around 0 especially for participants without T2D (Fig. 1A). Therefore, most non-cases are captured (specificity = 0.841), but many cases missed (sensitivity = 0.384) (Supplementary Table SS3 The AUC for incident cases (0.538) does not significantly differ from 0.5.

To evaluate whether PGS or FamRS do improve reclassification compared to a model already including several explanatory variables, NRI and IDI were additionally calculated. However, this was restricted to prevalent cases, since AUCs indicated a low predictive power for incident cases.

Adding the PGS to the baseline model could correctly increase T2D probability for 80% of the prevalent cases and decrease for 83% of non-cases, respectively (Supplementary Table S4). This results in an overall NRI of 1.261. The corresponding IDI was 0.328 (*p* = 1.02 × 10^–66^). These measures were nearly identical, when FamRS was also included to the baseline model.

Adding the FamRS to the baseline model could merely ameliorate prediction for non-cases by correctly down-shifting the probability for 85% of the non-cases (NRI = 0.361, *p* = 3.38 × 10^–9^), although overall NRI and IDI were still significant (IDI = 0.025, *p* = 0.0002). IDI was not significant any more, when FamRS was compared to a model already including the PGS (Supplementary Table S4).

The simultaneous addition of PGS and FamRS to the baseline model enhances the prediction for both prevalent cases (NRI_events_ = 0.579) and non-cases (NRI_non-events_ = 0.665), which leads to an overall NRI of 1.244 and IDI of 0.336 (*p* = 1.47 × 10^–66^).

## Discussion

In this study we found a strong association for both, a genome-wide polygenic score and family history, represented by a weighted family risk score, with the risk of prevalent and incident T2D in a population-based study from Southern Germany. Both scores were only slightly correlated with each other and were found to be independent predictors for T2D.

The PGS was found to be clearly right-shifted for prevalent and to a lesser extent incident T2D cases. The ORs were almost unaffected by any of the adjustments. While the finding of this association itself is in line with previous studies^10,20^, the extent of the effect size markedly differs. Mars et al.^21^ evaluated PGSs in different populations resulting in a pooled OR of 1.66 per 1 sd increase in mixed European populations, considering incident and prevalent cases jointly, while we found an OR of around 6 for prevalent and 1.66 in incident cases. In our study, the association was found to be non-linear, with a sharp increase of risk for the upper 20–25% of the distribution, which corresponds to the best discriminating cutoff. Also for these highest risk groups, a marked difference to the literature could be observed: For the top 5% vs. remainder, both Khera et al.^10^ and Mahajan et al.^20,22^ observed an OR of 2.75, while our study resulted in an adjusted OR of 47 for the same group. One explanation could be difference in prevalence (~ 2% in^10^, ~ 8% in KORA-F3) or definition/validation of Type 2 diabetes cases.

PGS was also found to be a valuable predictor for T2D with an AUC of 0.869 for prevalent and 0.613 for incident cases. It also significantly improves reclassification in addition to an already adjusted model and correctly shifts the probabilities for the majority of cases and non-cases to higher respectively lower values. Also here, reported AUCs for PGS were markedly lower (0.64–0.66^20^).

The FamRS was found to be significantly associated with both prevalent and incident T2D, supporting results of previous studies, which defined family history as any first-degree relative with T2D^8^ or by also taking into account the number of affected relatives^23^. The occurrence of T2D in either one of the parents was significantly associated with T2D risk with an OR of about 2.4 for prevalent and 1.6 for incident cases, even in the fully adjusted model. This is comparable to a Dutch study^24^, which found a HR of 2.2 for parental T2D, also after adjustment for lifestyle factors and obesity. When only looking at parental T2D, the risk discrimination between those with (strong) positive and very strong positive family risk would have been missed. A “very strong positive family risk” (FamRS > 2) occurs, if there are two or more T2D cases in a family with an age of onset before the age of 60. With such a measure, someone with very high familial risk might be identified, but a high fraction of individuals at risk might be missed, especially in small families. This is reflected in the prediction and reclassification measures. Although FamRS can significantly improve prediction, it is a better measure for correctly down-classifying non-cases than up-classifying cases.

However, family history scores taking into account family structure and age of onset, as the FamRS we used, have been shown to be more efficient than only looking at positive family history yes/no or even only disease status in parents^9,11,12,25^.

Unlike PGS, the effect size of FamRS is not stable across the different adjustment models for prevalent T2D. Although there is no difference from the unadjusted model to the model adjusting for age, sex, BMI and PA, adjustment for the PGS leads to an attenuation of the OR for FamRS risk categories, but it is still significantly associated. This means that a part of the information of FamRS is likely to be of genetic nature, while another part is independent from the PGS.

Cornelis et al.^26^ estimated by simulation and comparison with empirical data that about one third of the association of parental T2D with T2D risk is due to shared environment, while the remainder is due to shared genetics. This finding is supported by our data. Similar results have already been found for myocardial infarction^12,27^ and stroke^11^. For T2D, however, such data are sparse. Chatterjee et al.^28^ predicted—based on a theoretical model and observed SNP effect size distribution from GWAS—that family history, defined as presence of any affected first-grade relative, hardly improves polygenic risk prediction. In this theoretical framework, however, family history was assumed to represent only shared genetics. In UK-Biobank, however, including the family history of each of the relatives separately in a model improved prediction accuracy of polygenic risk scores^29^.

In this context, it should be noted that a recall bias cannot be excluded for the determination of the FamRS. It has been shown that both older individuals and those not affected from Diabetes were less accurate reporting the disease status of relatives than younger individuals and individuals with T2D^30^. This recall bias cannot play a role for incident cases, though, for which the FamRS was also shown to be significantly associated. In these incident models, the effect sizes for FamRS stay quite stable over different adjustment models. Especially for the very strong family risk group, there is also no decrease in effect size from the prevalent to the incident models. This is different for the PGS.

It has been shown that the heritability for T2D is twice as high in patients with age at onset 35–60 years (h^2^ = 0.69) compared to patients with onset up to 75 years (h^2^ = 0.31)^31^. Since the age of onset was about 10 years higher for incident cases in our study than for prevalent cases, the drop in effect sizes for the PGS can likely be explained by that. This assumption is further strengthened by the finding that effect sizes decrease for increasing 10y age-groups. However, confidence intervals overlap to a high degree, which advises against overinterpretations. However, previous studies^32,33^ have also found that polygenic scores play a greater role for those developing T2D at a younger age. For FamRS, however, there is only a drop in effect sizes for increasing age-groups, if it is unadjusted for the PGS. The drop cannot be observed any more, if it is adjusted for PGS, therefore getting rid of the “shared genetics” component. For higher age, the “shared environment” component, which also partly results in acquired habits and lifestyle, might thus play a bigger role in the FamRS-variable in older participants.

In conclusion, can and should PGS and FamRS be used to identify individuals with high T2D risk? Both scores have been shown to be quite independent from each other, and contribute to risk prediction in varying extent, depending primarily on age of onset. Even though a healthy lifestyle and especially having a normal body weight is beneficial for all in terms of T2D risk, independent of the genetic risk^34^, it has been shown that those being at high genetic risk defined by either high PGS^35^, but also high PGS and positive family history^36^ benefit most from adhering to a healthy lifestyle.

## Supplementary Information

Supplementary Information.

## Data Availability

The datasets generated and/or analysed during the current study are not publicly available due to restrictions from Helmholtz Zentrum München in accordance with the informed consent given by the study participants, but are available upon request from the corresponding author Prof. Lamina for any researcher based on a standard agreement on data provision within the KORA Research Platform using the digital tool KORA.PASST (https://helmholtz-muenchen.managed-otrs.com/external/c/requestprojectapplication).
