# Supplementary material for: A polygenic and family risk score are both independently associated with risk of type 2 diabetes in a population-based study
Source: Sci Rep. 2023 Mar 23;13:4805. doi: 10.1038/s41598-023-31496-w (PMC10036612; doi:10.1038/s41598-023-31496-w)

Supplemental material to

A polygenic and family risk score are both independently associated with risk of type 2 diabetes in a population-based study

Supplementary Methods

Definition and Calculation of the FamRS

The FamRS was calculated with an algorithm based on the information of a standardized interview.

For each parent and sibling, participant were asked the following question: “Does or did your … (e.g. father) have one of the following diseases?” with diabetes as one of the diseases in the list. No specification of diabetes subtypes was made. If the question for diabetes was answered with yes, it was followed-up by the question, if it was “before the age of 60”, “at the age of 60 or later” or “age unknown”.

To account for the age of onset, the following weights were included into the formula: Weight = 2 if age <60; Weight = 1 if age ≥60; Weight = 1.5 if age of disease onset is not known

The observed values (O) for each participant were calculated by taking the sum of the weights for all first-grade relatives. The expected values (E) were derived from the mean values of the weights for father, mother, brothers, and sisters for each 10-year age group, respectively. Since KORA F3 is a population-based study, these mean values were considered to be appropriate reference values. The FamRS was then calculated as suggested by Williams and colleagues[1]:

If $|O - E| > 0.5$ then $\text{FamRS} = \frac{|O-E|-0.5}{\sqrt{E}} \times \frac{|O-E|}{O-E}$

or if $|O - E| \leq 0.5$, then FamRS=0. If FamRS is ≥1.0 with only one affected person in the family, FamRS is set to 0.99.

The following categorizations were recommended by Williams et al. [1] .: average (FamRS≤0.5), positive (>0.5 to 1.0), strong positive (>1.0 to 2.0), and very strong positive family history (>2.0). These categories can be interpreted like this: average refers to no events in an average or small sized family or one event at an higher age in a large family; positive refers to one event at any age in families of small or average size or one early event in large families; strong positive refers to one early or two events at any age; and very strong positive refers to two or more events at an early age.

[1] Williams RR, Hunt SC, Heiss G, Province MA, Bensen JT, Higgins M, et al. Usefulness of cardiovascular family history data for population-based preventive medicine and medical research (the Health Family Tree Study and the NHLBI Family Heart Study). Am J Cardiol. 2001 Jan;87(2):129–35.

Supplementary Tables

Table S1: Absolute and relative amount of participants with T2D in each age group (age at baseline), in subgroups of the PGS and FamRS

Subgroup	Sample size per subgroup (available for baseline analysis)	Prevalent T2D cases	Sample size per subgroup (available for follow-up analysis)	Incident T2D cases
**Age-groups**				
35-44	617	6 (1.0%)	549	13 (2.4%)
45-54	715	18 (2.5%)	635	34 (5.34)
55-64	733	75 (10.2%)	597	46 (7.7%)
65-74	651	97 (14.9%)	496	45 (9.1%)
75-84	355	60 (16.9%)	258	25 (9.7%)
**Percentile groups of the PGS**				
Lower 80% of PGS	2456	65 (2.6%)	2173	121 (5.6%)
Upper 20% of PGS	615	191 (31.1%)	362	42 (11.2%)
Upper 10% of PGS	308	155 (50.3%)	126	21 (16.7%)
Upper 5% of PGS	154	107 (69.5%)	35	9 (25.7%)
**FamRS categories**				
Average family risk	2582	175 (6.8%)	2175	126 (5.8%)
positive and strong positive family risk (FamRS 2)	343	45 (13.1%)	265	21 (7.9%)
very strong positive family risk (FamRS 3)	125	32 (25.6%)	80	14 (17.5%)

Table S2: Results of Logistic regression models of the effect of "one of the parents having had T2D" on risk of prevalent and incident diabetes.

	Outcome: Prevalent T2D			Outcome: Incident T2D		
	OR	CI (95 %)	p-value	OR	CI (95 %)	p-value
Unadjusted	2.14	[1.64 – 2.79]	2.13×10^{-8}	1.49	[1.05 – 2.08]	0.0217
Adjusted for Age + Sex + BMI + Physical activity	2.68	[1.99 – 3.62]	4.22×10^{-11}	1.67	[1.16 – 2.39]	0.0052
Adjusted for Age + Sex + BMI + Physical activity + PGS	2.41	[1.66 – 3.51]	4.26×10^{-6}	1.64	[1.13 – 2.35]	0.0080

Table S3: Area under the curve (AUC) for PGS and FamRS and best discriminating thresholds for prevalent and incident cases.

Parameter	AUC	95% CI for AUC using DeLong method	Best discriminating threshold	Specificity for best threshold	Sensitivity for best threshold
As predictor for prevalent diabetes					
PGS	0.869	[0.842; 0.896]	0.016	0.802	0.820
FamRS	0.617	[0.579; 0.655]	0.109	0.840	0.377
As predictor for incident diabetes					
PGS	0.613	[0.565; 0.657]	-0.053	0.599	0.604
FamRS	0.539	[0.490; 0.587]	0.009	0.821	0.280

Table S4: Results of Continuous Net Reclassification index (NRI) overall and separated for events and non-events and Integrated Discrimination Improvement (IDI); all measures are given with 95% CI and corresponding p-values

Base Model	NRI			NRI events				NRI nonevents				IDI	
	Index	Lower and upper limit of 95% CI	p-value	Increase for events*	Index	Lower and upper limit of 95% CI	p-value	Decrease for non-events†	Index	Lower and upper limit of 95% CI	p-value	Index	p-value
**Adding PGS**													
Age,sex,BMI, PA	1.261	1.159 1.364	4.42×10^{-128}	0.801	0.602	0.503 0.700	8.09×10^{-33}	0.830	0.660	0.632 0.687	$<1 \times 10^{-300}$	0.328	1.02×10^{-66}
Age,sex,BMI, PA, FamRS	1.273	1.172 1.375	1.42×10^{-132}	0.810	0.619	0.522 0.717	2.59×10^{-35}	0.827	0.654	0.626 0.682	$<1 \times 10^{-300}$	0.312	3.08×10^{-62}
**Adding FamRS**													
Age,sex,BMI, PA	0.361	0.241 0.480	3.38×10^{-09}	0.324	-0.352	-0.469 -0.236	3.32×10^{-09}	0.856	0.713	0.687 0.739	$<1 \times 10^{-300}$	0.025	0.000189
Age,sex,BMI, PA, PGS	0.312	0.194 0.429	2.11×10^{-07}	0.304	-0.393	-0.507 -0.278	1.93×10^{-11}	0.852	0.704	0.678 0.731	$<1 \times 10^{-300}$	0.005	0.13548
**Adding PGS and FamRS**													
Age,sex,BMI, PA	1.244	1.139 1.350	1.83×10^{-118}	0.789	0.579	0.477 0.681	6.45×10^{-29}	0.833	0.665	0.638 0.693	$<1 \times 10^{-300}$	0.336	1.47×10^{-66}

* Proportion of participants with Type 2 Diabetes, for whom probability of having Type 2 diabetes was correctly increased after adding PGS, FamRS or both; † Proportion of participants without Type 2 Diabetes, for whom probability of having Type 2 diabetes was correctly decreased after adding PGS, FamRS or both

Supplementary Figures

Figure S1: Proportion of prevalent T2D cases for number of relatives affected by diabetes

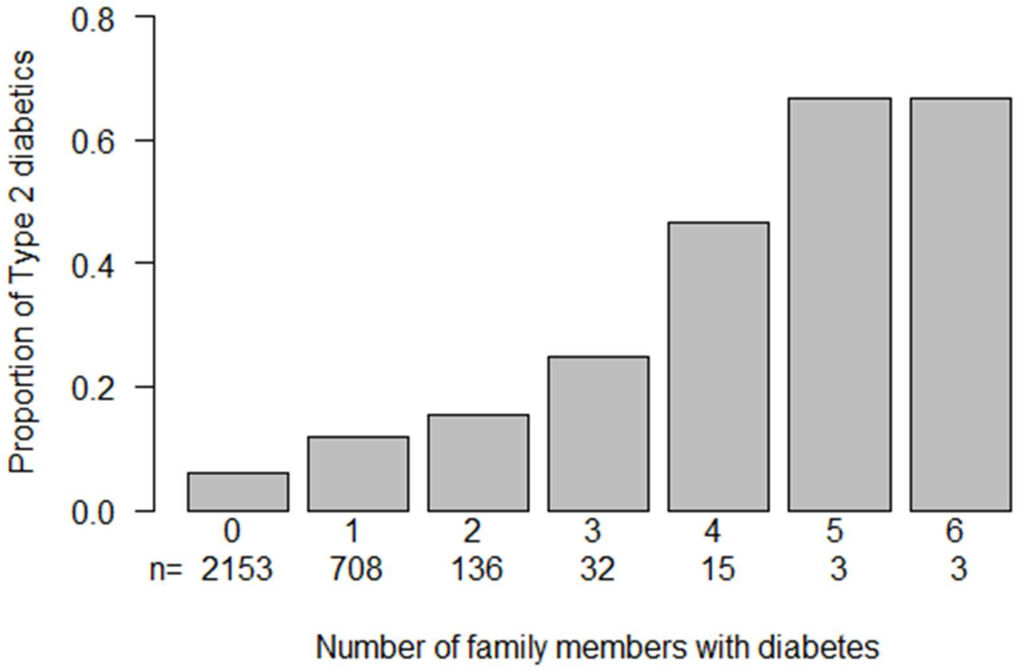

Figure S2: Density plot showing the FamRS distribution in individuals who did not (lightblue, panel A) or did (pink, panel B) develop diabetes during the time between the KORA F3 study and the follow-up in 2016 (incident cases); the lines depict the thresholds of the FamRS categories: FamRS > 0.5: positive family risk (blue line), FamRS > 1: strong positive family risk (purple), FamRS > 2: very strong positive family risk (red line).

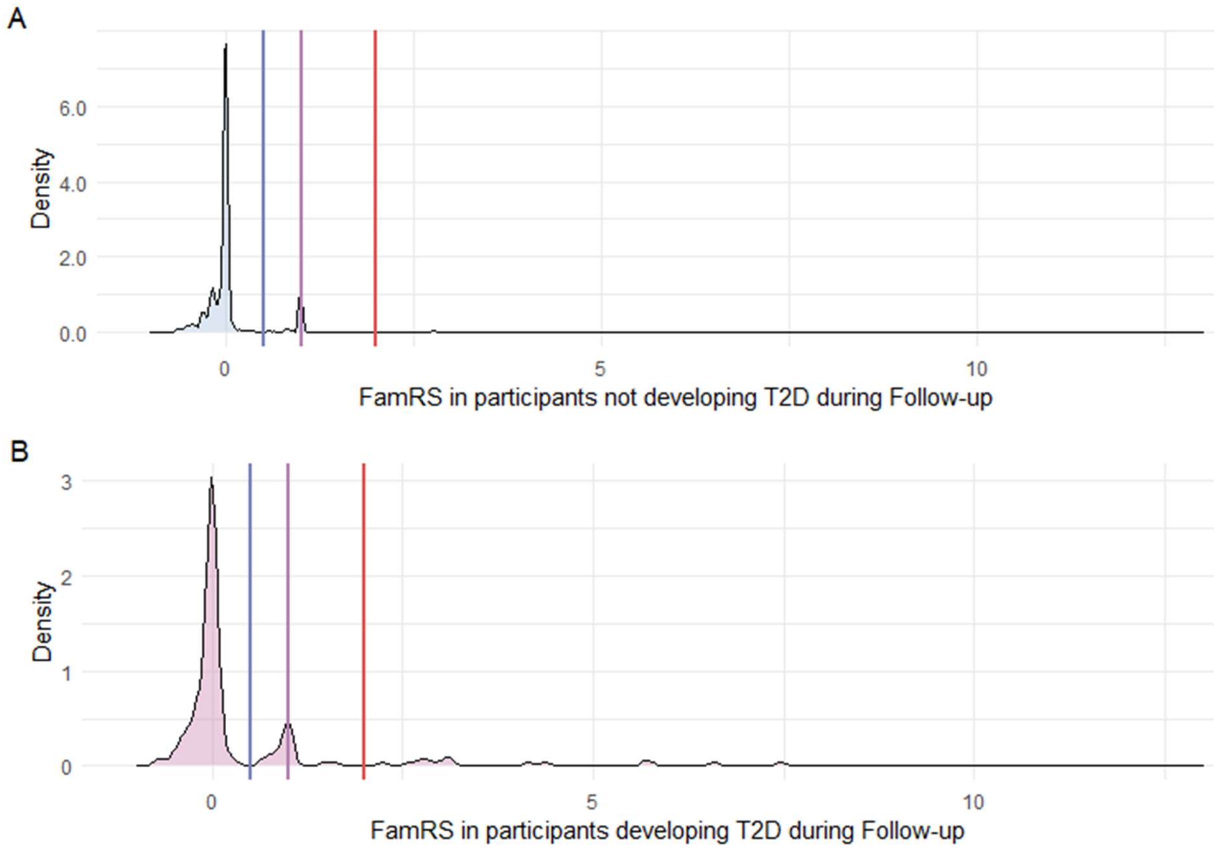

Figure S3: Boxplots of the FamRS distribution of participants with T2D in the age groups 35-44, 45-54, 55-64, 65-74 and 75-84 (at baseline) in comparison to the FamRS in participants without T2D.

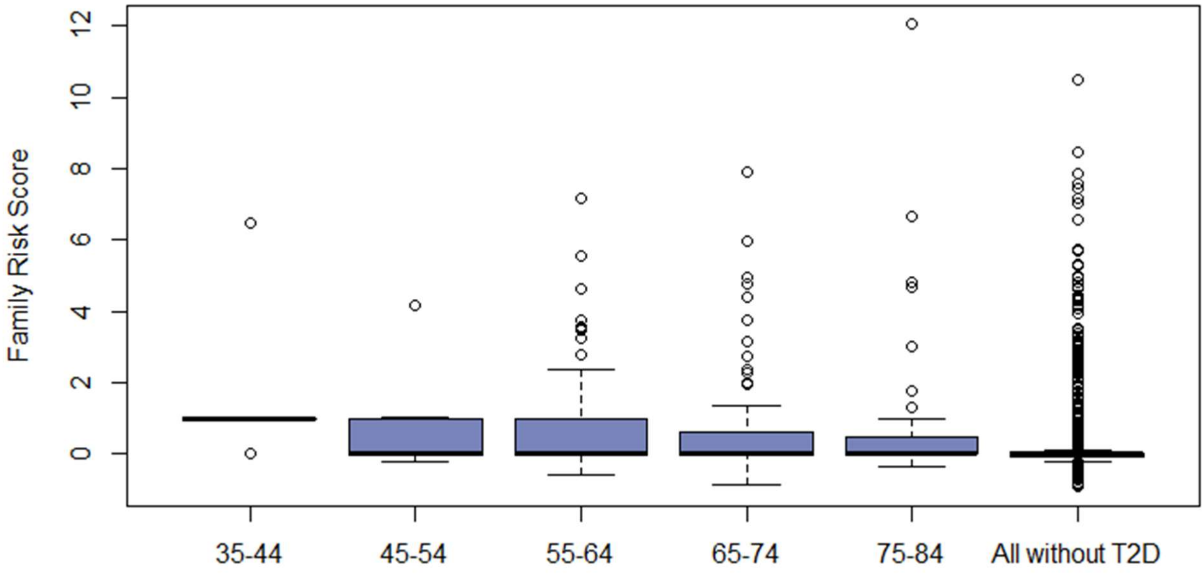

Figure S4: Comparison of the distribution of the PGS in the following populations (from top to bottom): South Asian, KORA F3, European, East Asian, American and African population

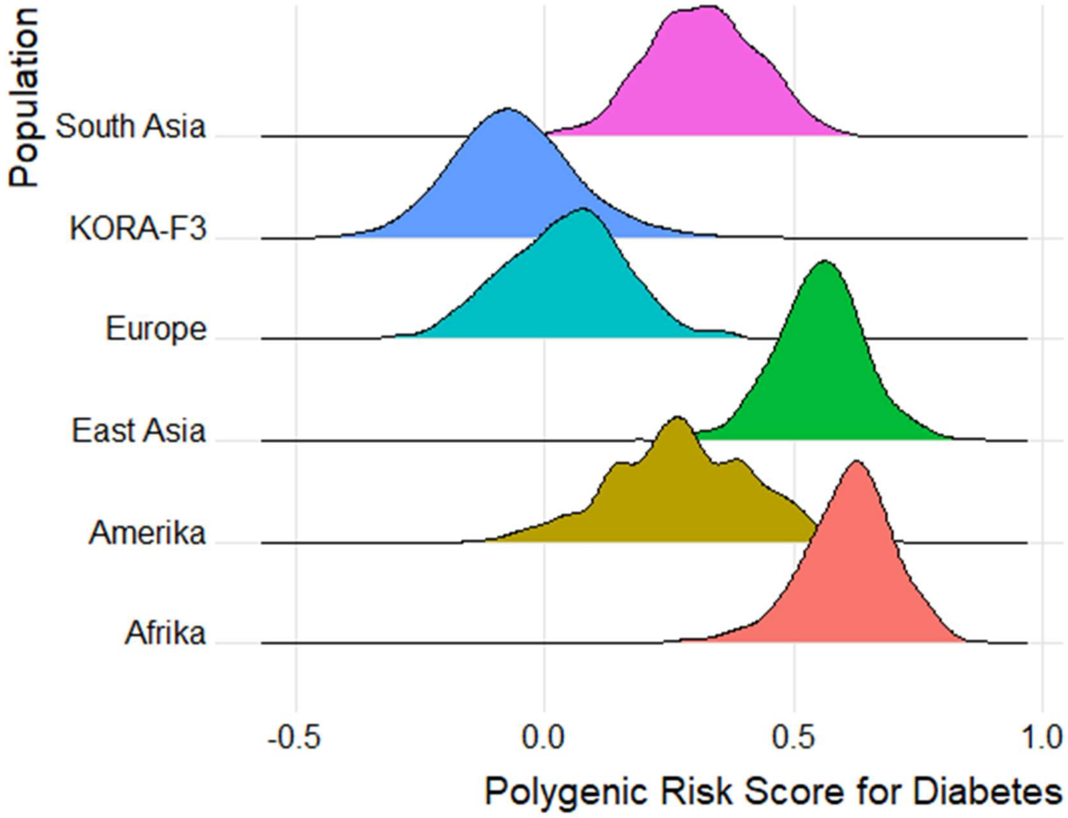

Figure S5: Density plot showing the PGS distribution in individuals who did (pink) versus did not (lightblue) develop diabetes during the time between the KORA F3 study and the follow-up in 2016 (incident cases).

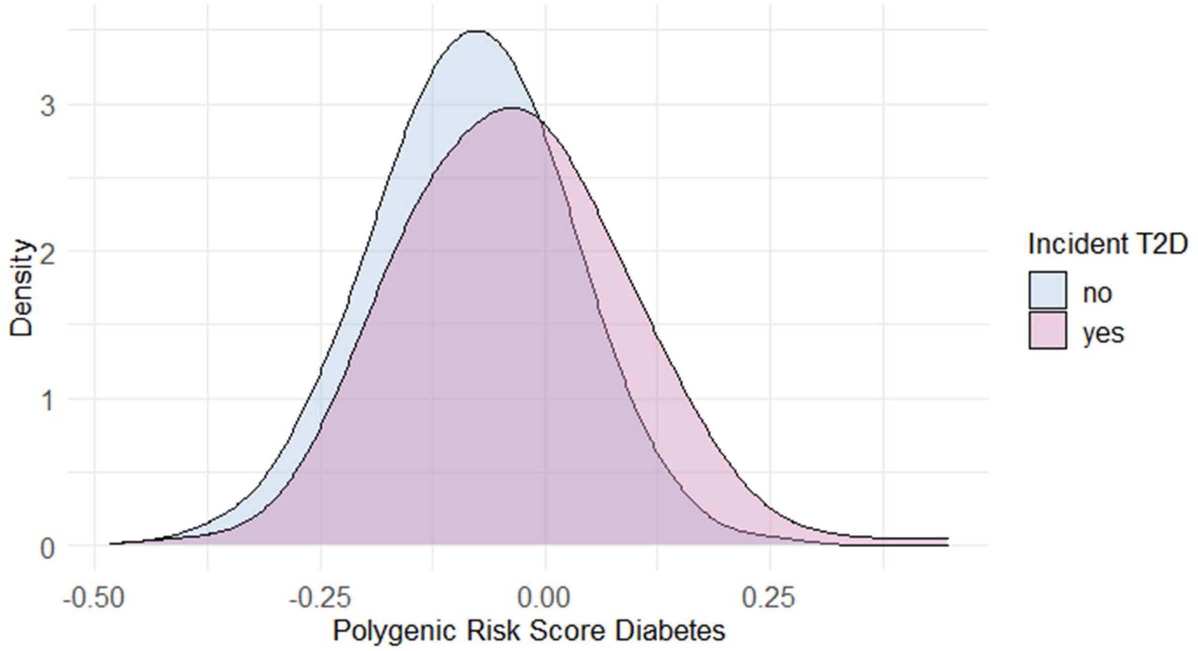

Figure S6: Boxplots of the PGS distribution of participants with T2D in the age groups 35-44, 45-54, 55-64, 65-74 and 75-84 (at baseline) in comparison to the PGS in participants without T2D.

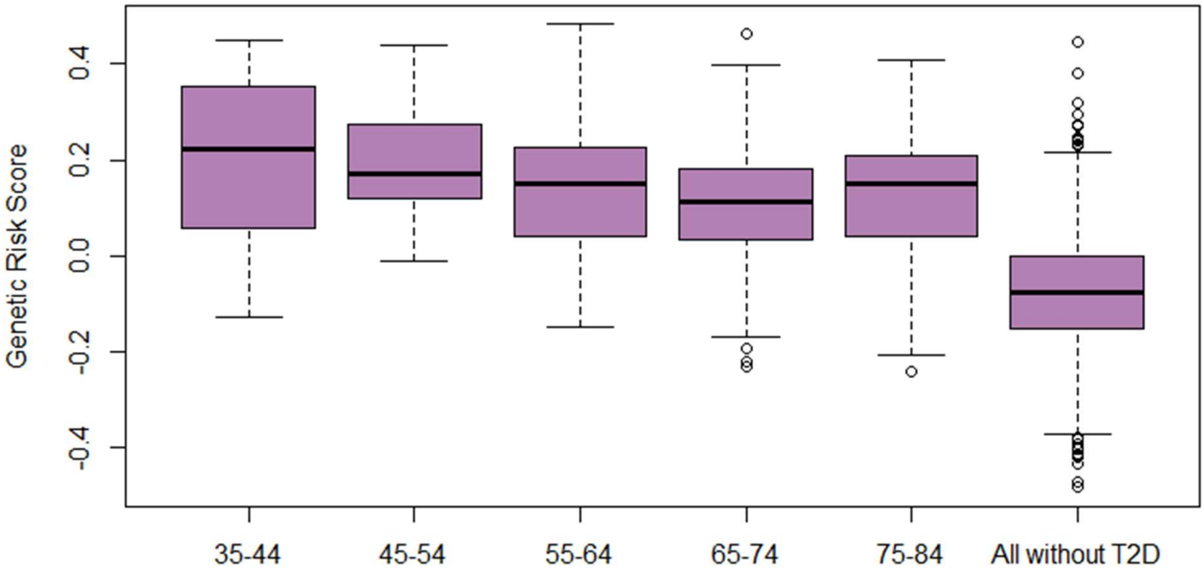

Figure S7: Prevalence of Type 2 Diabetes per 100.000 in the following percentile-groups of the PGS: 0-5%, 5-10%, 10-15%, 15-20%, 20-25%, 25-30%, 30-35%, 35-40%, 45%, 45-50%, 50-55%, 55-60%, 60-65%, 65-70%, 70-75%, 75-80%, 80-85%, 85-90%, 90-95%, 95-100% (dot is placed in the middle of the 5%-range).

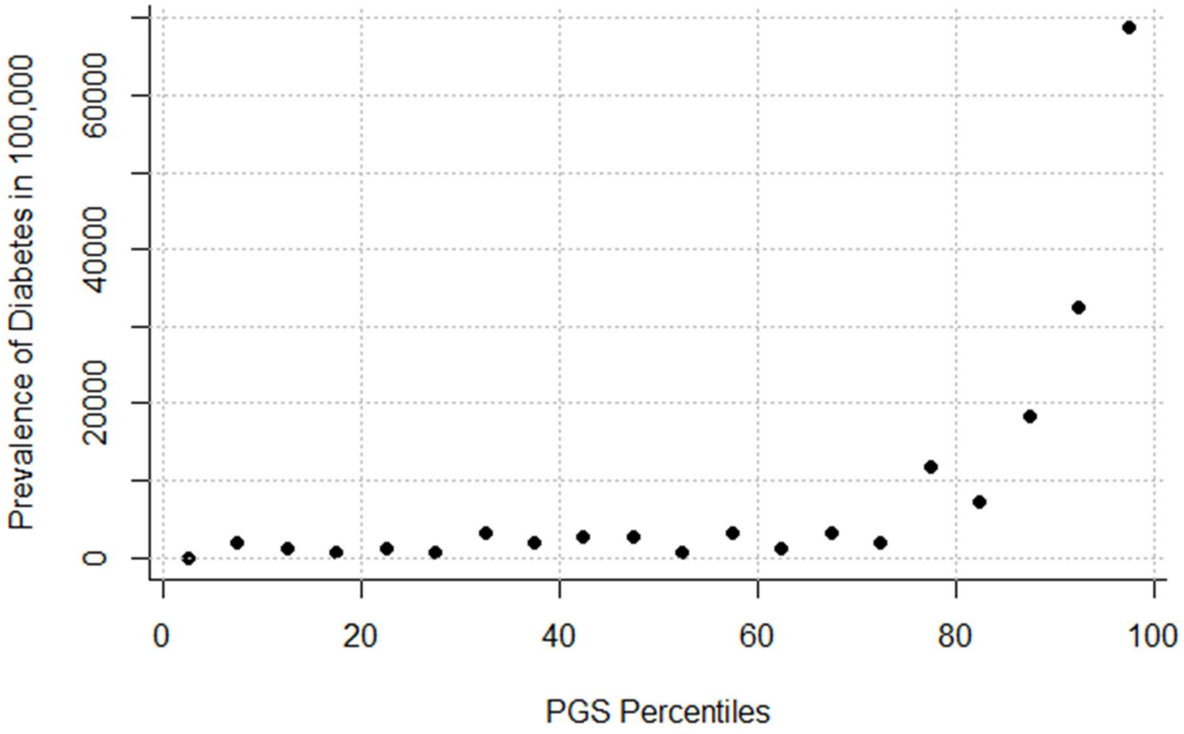

Figure S8: PGS distribution depending on the amount of family members affected by diabetes

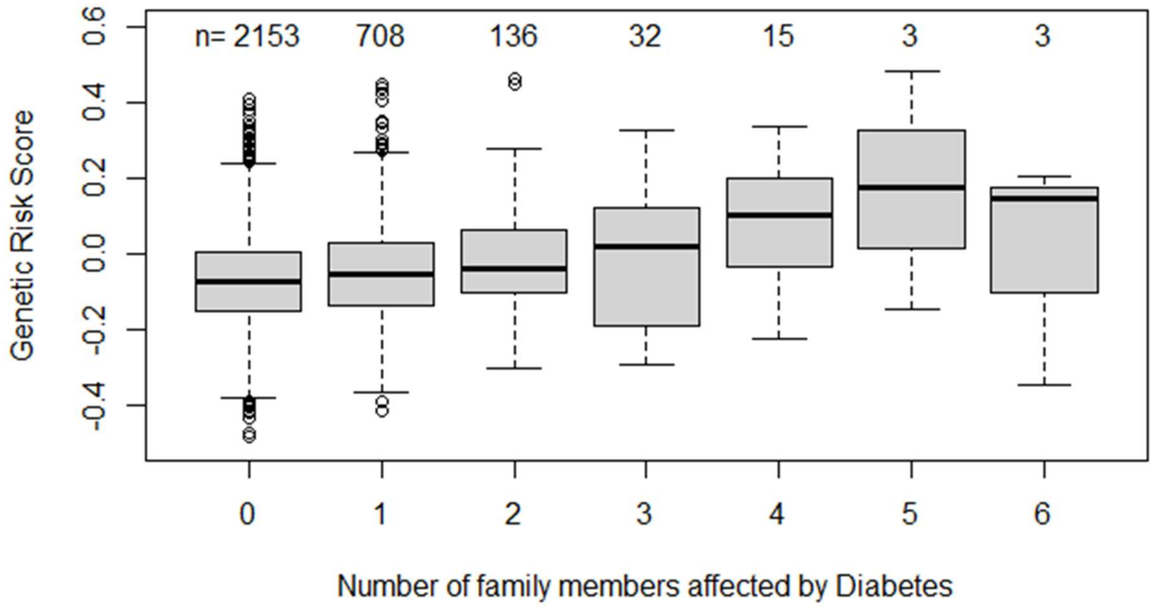

Figure S9: Results of logistic regression models of PGS and FamRS on prevalent T2D cases in 10y age-groups; A) ORs for "above average family risk" (FamRS ≥ 0.5), stratified in 10y age-groups; B) ORs for PGS in 1sd, stratified in 10y age-groups; Results are given for both unadjusted models (black lines) as well as PGS and FamRS mutually adjusted for each other (blue lines).

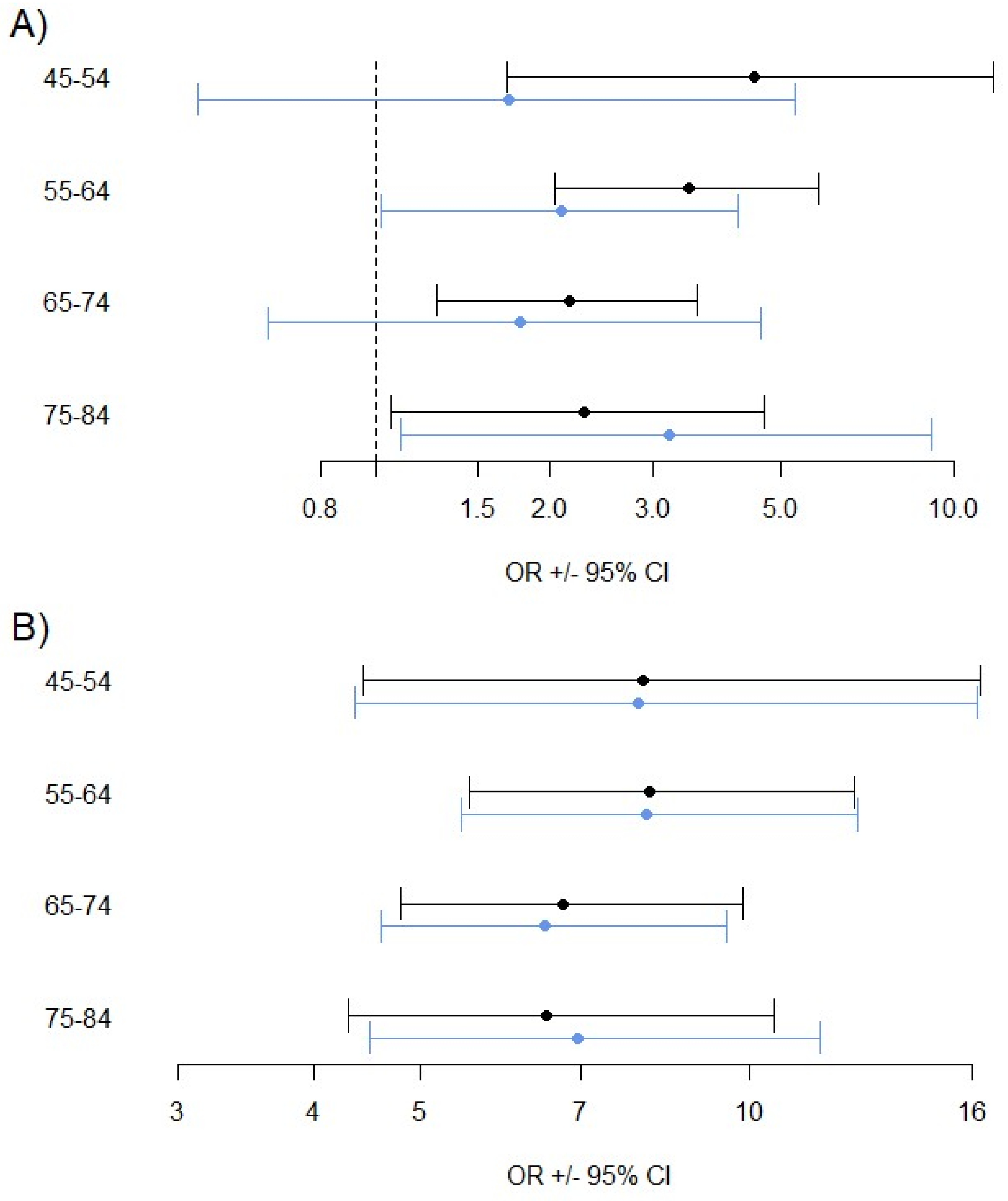

Supplement: Supplementary file 1 — Supplementary Information. [file 41598_2023_31496_MOESM1_ESM.pdf]
